# Exposure of Polystyrene Nano- and Microplastics in Increasingly Complex In Vitro Intestinal Cell Models

**DOI:** 10.3390/nano15040267

**Published:** 2025-02-11

**Authors:** Kristen A. Marcellus, David Prescott, Michal Scur, Nikia Ross, Santokh S. Gill

**Affiliations:** Regulatory Toxicology Research Division, Bureau of Chemical Safety, Health Products and Food Branch, Health Canada, Ottawa, ON K1A 0K9, Canada

**Keywords:** microplastics, nanoplastics, intestinal cells, barrier integrity, polystyrene

## Abstract

With the rise in global plastic production and the presence of plastic waste in the environment, microplastics are considered an emerging environmental contaminant. Human exposure and the impact of microplastics on human health are not well studied. Recent studies have observed the presence of microplastics in human tissues and several studies have noted toxicity in in vitro and in vivo mammalian models. We examined the impact of polystyrene nano- and microplastics in increasingly complex intestinal cell models. Using an undifferentiated Caco-2 mono-culture model, we assessed particle association, cytotoxicity, and particle clearance/retention, whereas in differentiated mono- and tri-culture transwell models, we assessed membrane integrity and particle translocation. Only 50 nm and 500 nm particles were internalized in the undifferentiated cells; however, no signs of cellular toxicity were observed at any concentrations tested. Additionally, polystyrene particles had no impact on barrier integrity, but the 50 nm particles were able to cross to the basolateral side, albeit attenuated in the tri-culture model that had a mucus layer. This study reduced some of the variability common to MNPL testing across various in vitro models, but further testing is needed to fully understand the potential effects of human MNPL exposure.

## 1. Introduction

Global plastic production has exceeded 300 million tons per year and plastic waste has become an environmental issue [1,2,3,4,5,6,7]. Microplastics (MPs) are defined as plastic particles with a diameter of 0.1 μm–5 mm, while nanoplastics (NPs) are <0.1 μm [1,3,5,8,9]. Small-sized plastic particles purposefully manufactured for a specific function, such as microbeads found in personal care products, are called primary microplastics [3,6,10,11,12], whereas secondary microplastics are generated by the degradation or fragmentation of larger plastic waste released into the environment by UV radiation, microbial degradation, and physical forces [1,3,5,6,8,11,12,13,14,15].

MNPLs (micro- and nanoplastics) can be detected analytically throughout the environment, including both terrestrial and marine environments, and humans may be exposed to MNPLs [7,16]. Recently, MPs have been reported in human stool samples and colon, intestine, and various other tissues [17,18,19,20,21,22,23,24,25,26,27,28,29,30,31,32]. Human exposure to MNPLs may occur through the ingestion of food or drinking water [5,8,33] and via the inhalation of indoor and outdoor air [1,5,34]. MPs have been reported in multiple aquatic species consumed by humans such as fish, lobsters, mussels, oysters, sea cucumbers, and scallops, as well as other food commodities such as salt, honey, sugar, seaweed, milk, and drinking water [1,6,7,8,12,35]. This raises the need for data on absorption and potential adverse health effects after MNPL ingestion.

The first site of exposure after MNPL ingestion is the gastrointestinal tract (GIT), with the gut barrier, composed of the intestinal epithelium and mucus, acting as both a physical and chemical barrier [36]. MNPL uptake is variable, with different parts of the GIT receiving different exposure levels, as more MPs have been detected in the proximal part of the small intestine [37]. Recent studies examining the effect of MPs and NPs using in vivo mammalian models have shown an accumulation of MPs and NPs in the gut, liver, kidney, heart, and stomach wall of rodents [12,38,39,40,41]. Reported pathological changes in the gut of mice consisted of reduced mucus secretion [41,42,43], gut barrier dysfunction [39,41,44], intestinal inflammation [41,45,46], and gut microbiota dysbiosis [39,41,42,43,44,46].

A small percentage of ingested MNPLs < 150 µm (<0.3%) are thought to be able to translocate through the gut epithelium and enter the circulatory system, with only very small MNPLs < 1.5 µm expected to accumulate in various organs [5,33,35,40,47,48,49]. Importantly, numerous studies with experiments on animals have shown that MP/NP particles can be absorbed through the intestinal barrier and enter systemic circulation [1,9,33,50]. Ingested particles may accumulate locally in the intestine; thus, it is of mechanistic importance to assess the in vitro uptake and effects of MP/NPs in intestinal cells, thereby reflecting the primary target exposed to these particles in vivo [1,51].

Several studies have examined the effect of MPs and NPs on human cells in cultures with mixed results [14,48,50,52,53,54,55,56,57,58]. Observed results from different studies are varied and contradictory due to different research models, MP/NP diversity, and the lack of a standardized detection and quantification methodology [1,59]. There are serval factors to account for in MP/NP diversity and how it can impact toxicity. One such factor is polymer composition [1]. The most common plastic polymers detected in the environment are polystyrene, polypropylene, and high-density and low-density polyethylene [5,15,58,60,61]. In addition to polymer type, the size and shape of MP/NPs can impact their toxicity. Previous studies have shown that the size of MP/NPs plays a critical role in the absorption and toxicity of these particles [5]. Typically, cells internalize small particles to a greater extent than larger ones [62]. Smaller particles are internalized through endocytosis or passive uptake processes, whereas larger particles generally require phagocytosis [1]. Another factor in cellular uptake is the dose and exposure duration, where a higher dose and chronic exposure can lead to higher uptake, resulting in greater toxicity risk [1].

Due to the associated complexity of MP/NP interaction in human biological systems, we chose to investigate the effects of MP/NPs on increasingly complex in vitro human gut models. Caco-2, a well-established model for human small intestinal enterocytes, spontaneously differentiates and forms a monolayer, which mimics the intestinal barrier when used in a transwell system [63]. The addition of HT29-MTX-E12 goblet-like cells that secrete mucin and differentiated THP-1 macrophages better simulates the multicellular environment of the human intestine [64,65,66]. We used three intestinal epithelial models: an undifferentiated Caco-2 mono-culture, a differentiated Caco-2 mono-culture transwell model, and a tri-culture transwell model, and utilized the advantages of each type of culture to gain insight into the effects of MNPL exposure. Briefly, in undifferentiated Caco-2 cells, we focused on elucidating uptake and toxicity at the cellular level, based on the size and concentration of the PS particles after 24 h and 7 days of exposure. In the transwell systems, we examined barrier integrity and particle translocation across the monolayer.

## 2. Materials and Methods

### 2.1. Polystyrene Microspheres

Fluoresbrite^®^ YG and Polybead^®^ polystyrene microspheres were obtained from Polysciences (Polysciences Inc., Warrington, PA, USA). The 50 nm microspheres were packaged in a 2.5% (*w*/*v*) aqueous suspension at a concentration of 3.64 × 10^14^ particles/mL. These beads had a coefficient of variation of 15%. The 500 nm and 1 µm microspheres were also packaged in a 2.5% (*w*/*v*) aqueous suspension at a concentration of 3.64 × 10^11^ particles/mL and 4.55 × 10^10^ particles/mL, respectively, and both had a coefficient of variation of 3%. The microspheres were vortexed for 30 s and diluted in complete media at final concentrations of 0.01 µg/mL–100 µg/mL. The mean particle size and particle distribution were measured in distilled ultrapure water and complete Dulbecco’s Modified Eagle Medium (DMEM) (Table A1) using a Zetasizer (PN3702, Malvern Panalytical, Malvern, UK).

### 2.2. Cell Culture

All cells were routinely checked for mycoplasma using the MycoAlert^®^ Mycoplasma Detection Kit (Lonza, Morristown, NJ, USA). All cell cultures were incubated at 37 °C and 5% CO_2_.

### 2.3. Mono-Culture

The human colon adenocarcinoma cell line Caco-2 was obtained from the American Type Culture Collection (HTB-37) and cultured in Eagle’s Minimum Essential Medium (EMEM; Wisent Bioproducts, Saint-Jean-Baptiste, QC, Canada) supplemented with 20% fetal bovine serum (FBS; Wisent Bioproducts), 1% penicillin/streptomycin (ThermoFisher, Burlington, ON, Canada), and 1% Glutamax (ThermoFisher). The medium was supplemented with MycoZap™ Prophylactics (Lonza).

### 2.4. Mono-Culture Transwell

Caco-2 cells were cultured in DMEM (ThermoFisher) supplemented with 10% FBS (Wisent Bioproducts or ThermoFisher), 1% Glutamax, and 1% MEM non-essential amino acids (Millipore Sigma, Oakville, ON, Canada). The cells were plated at a seeding density of 4 × 10^4^ cells/well in a polyester Transwell™ membrane (6.5 mm, 3.0 µm pore size; Corning, Corning, NY, USA). The cells were cultured for 21–25 days, until the transepithelial electrical resistance (TEER) was greater than 1000 Ω.

### 2.5. Tri-Culture Transwell

Caco-2 cells were cultured in DMEM supplemented with 10% FBS, 1% Glutamax, and 1% MEM non-essential amino acids. Human mucus-secreting goblet-like HT29-MTX-E12 cells (Millipore Sigma) were cultured in DMEM supplemented with 10% FBS, 1% Glutamax, and 1% MEM non-essential amino acids. Leukemic monocyte THP-1 (ATCC TIB-202) cells were cultured in Roswell Park Memorial Institute (RPMI) 1640 (ThermoFisher) supplemented with 10% FBS and 1% Glutamax. The Caco-2 and HT29-MTX-E12 cells were plated at a cell density of 2.4 × 10^4^ cells/well in a 9:1 ratio, respectively, in Transwell™ inserts. Upon reaching confluence, the cells were cultured for 21 days to allow for enterocyte differentiation, with frequent media changes. The THP-1 cells were seeded at 2.4 × 10^5^ cells/well in a 24-well plate 5 days prior to the 21st day of differentiation. The THP-1 cells were differentiated for 65 h with 20 ng/mL phorbol 12-myristate 13-acetate (PMA; Millipore Sigma); this was followed by a change to medium containing no PMA for 24 h. Transwell™ inserts were combined with the wells containing THP-1 to create the tri-culture and left to establish for 24 h. Only wells with a TEER greater than 1000 Ω were used in experiments.

### 2.6. Cytotoxicity Assay

CyQuant™ MTT Cell Viability Assay (ThermoFisher) was used according to the manufacturer’s quick protocol instructions. Cells were plated on black 96-well plates (Corning or ThermoFisher) 24 h prior to PS particle exposure. The cells were treated with non-fluorescent microspheres for 24 h or 7 days at concentrations ranging from 0 to 100 µg/mL, with new microbeads in fresh media every 2–3 days. Absorbance was read at 540 nm (Cytation 3; Agilent Technologies, Santa Clara, CA, USA). The viability of the control cells (treated with dH_2_O and not exposed to microspheres) was set to 100%.

### 2.7. Flow Cytometry

Cells were plated on 6- or 12-well plates (Greiner Bio-One, Monroe, NC, USA) 24 h before microsphere addition. The cells were exposed to fluorescent microspheres for 24 h. For cellular membrane association and uptake quantification, cells were collected after 24 h of exposure to MPs, whereas cells used for the clearance assay were washed with PBS after 24 h of exposure and left in regular culture conditions for 7 more days before collection. The cells were washed with PBS and collected using tryspsin/EDTA (ThermoFisher) at 37 °C. The cells were resuspended in staining buffer (5% FBS in PBS) with SYTOX™ blue dead cell stain (ThermoFisher) and analyzed using the BD LSRFortessa™ flow cytometer (BD, Franklin Lakes, NJ, USA). The fluorescence of each cell was assessed after exposure to nano- and micro-YG-PS-beads. Control cells, not exposed to microbeads, were used to set the threshold of the fluorescent signal. Data analysis was performed using FlowJo™ v10.8 software (BD Life Science, Ashland, OR, USA).

### 2.8. Immunofluorescence

Cells were plated on 8-chambered slides (ThermoFisher) and treated with PS particles for 24 h. The cells were washed 3 times with PBS before being fixed with 4% formaldehyde in PBS with a pH of 7.4 for 15 min and then permeabilized with 0.5% Triton X-100 in 1X PBS for 15 min. The cells were then blocked with 10% fetal bovine serum (FBS) for 2 h at room temperature. The cells were incubated with Alexa Fluor™ 594 Phalloidin (A12381, ThermoFisher) diluted in 1% FBS and 0.1% Triton X-100 for 2 h at room temperature. The cells were washed 3× with PBS and mounted on ProLong Diamond Antifade mounting media containing DAPI for staining the nuclei (ThermoFisher). The cells were visualized by microscopy at 40× using the Cytation C10 (Agilent Technologies) microscope.

Tri-culture transwells were incubated with PS particles for 24 h. The supernatant was aspirated and the transwells were washed gently 3 times with PBS before being fixed by immersion in Carnoy’s solution (VWR, Allentown, PA, USA) for 1 h. Following fixation, the transwells were submerged in 70% ethanol until they were embedded in paraffin blocks. The blocks were sectioned using a microtome with a 6 µm thickness. Sections were deparaffinized and rehydrated via sequential baths in toluene and a series of decreasing concentrations of isopropanol. The sections were washed in water and blocked with 3% normal goat serum (ThermoFisher) in PBS for 1 h at room temperature.

For the wheat-germ agglutinin-staining of mucus, sections were incubated with Alexa-Fluor 594-conjugated wheat-germ agglutinin (W11262, ThermoFisher) at a concentration of 1:300 in PBS with 3% normal goat serum and 0.1% tween-20 (Sigma Millipore) for 2 h at room temperature. After incubation, the slides were washed 3 times for 5 min in PBS and mounted using Prolong Diamond antifade mounting media with DAPI (ThermoFisher). The slides were imaged at 40× using Cytation C10 (Agilent Technologies).

### 2.9. TEER Measurements

Cells were cultured as described above and exposed to fluorescent microspheres for 24 h on either the apical side or the basolateral side of the transwell system. Cell barrier integrity was determined using the TEER as measured using an epithelial volt–ohm meter (EVOM2; World Precision Instruments, Sarasota, FL, USA). An insert without cells was measured for background resistance and used to correct the TEER values. The resistance values of the mono-culture and tri-culture were calculated by subtracting the TEER value of the blank insert from the TEER value measured in each well and multiplying it by the surface area of the insert (Ω×cm^2^), before normalizing to control the values.

### 2.10. Translocation

Cell culture medium in the basolateral compartment from culture models exposed to fluorescent microspheres in the apical compartment was collected and analyzed for the fluorescent signal using a plate reader (Synergy H1 or Cytation 10; Agilent). For the quantification of particle translocation, standard curves for each particle size were used. Dilutions were prepared in cell culture media.

### 2.11. Statistical Analysis

All experiments were performed with 3 or more biological replicates unless stated otherwise. Data are presented as the mean ± standard error of the mean. Data were analyzed with one-way analysis of variance (ANOVA) or two-way ANOVA followed by Dunnett’s multiple comparison post hoc test in GraphPad10 Software (Boston, MA, USA). Significance was set at *p* ≤ 0.05.

## 3. Results

### 3.1. Undifferentiated Mono-Culture

#### 3.1.1. Cytotoxicity

**Figure 1:**
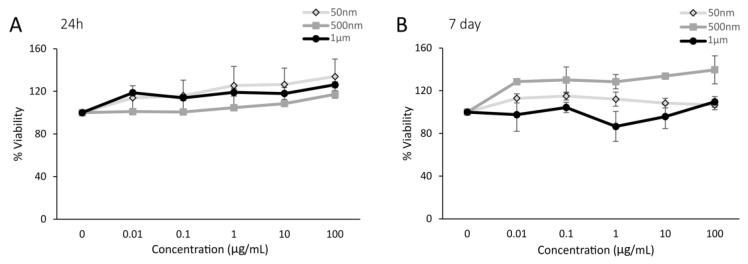
Polystyrene particles caused no cytotoxicity in undifferentiated Caco-2 cells. Cell viability was measured in Caco-2 cells exposed to polystyrene particles (50 nm, 500 nm, and 1 µm) at concentrations of 0–100 µg/mL for 24 h and 7 days. Cell viability was assessed by an MTT assay. The quantification of cell viability is presented as a percentage relative to the CTL (unexposed cells). (**A**) Caco-2 cells after 24 h of exposure. (**B**) Caco-2 cells after 7 days of exposure. Data are the mean ± SEM from three independent experiments (n = 3). One-way ANOVA with Dunnet’s post hoc test was used with *p* > 0.05 indicating N.S.

To determine the impact of PS micro- and nanoplastic particles on the cell viability of Caco-2 cells, an MTT assay was performed. After 24 h post-exposure to PS-NPs and -MPs, no decrease in cell viability was observed, as seen in Figure 1A. Similarly, there was no significant reduction in cell viability for Caco-2 cells exposed to 50 nm, 500 nm, and 1 µm PS particles for 7 days (Figure 1B).

#### 3.1.2. Cellular Association

**Figure 2:**
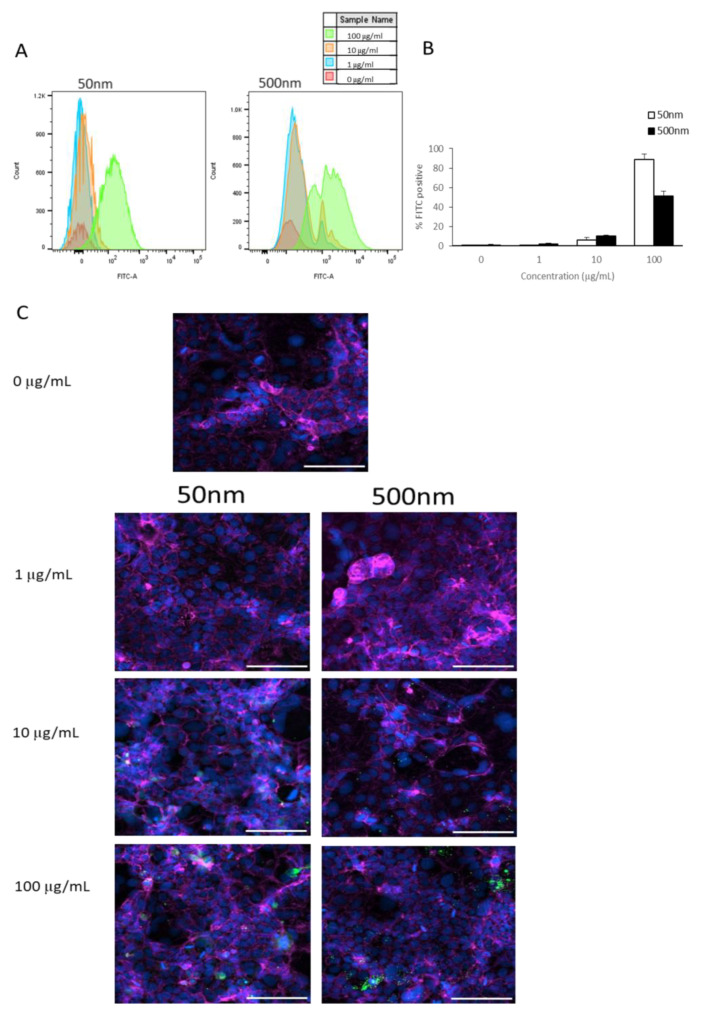
Caco-2 internalization of PS-NPs and PS-MPs. Cellular membrane association and uptake was analyzed by flow cytometry after cells were exposed to YG-PS-beads for 24 h. (**A**) Representation of mean fluorescent intensity peaks of undifferentiated Caco-2 exposed to YG-PS-beads. (**B**) Quantification of percentage of Caco-2 that internalized YG-PS-NPs and -MPs (n = 3). (**C**) Representative images of Caco-2 cells exposed to 50 nm and 500 nm YG-PS-beads for 24 h. Scale bar = 100 µm. Cell nuclei were stained with DAPI (blue); phalloidin staining is shown in magenta and YG-PS-beads are green.

**Figure A1:**
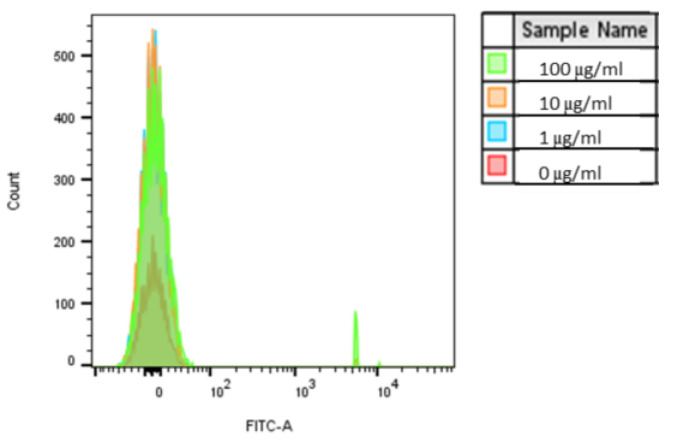
Caco-2 internalization of 1 µm PS-MPs. Uptake was analyzed by flow cytometry after cells were exposed to YG-PS-beads for 24 h. Representation of mean fluorescent intensity peaks (n = 1).

Flow cytometry was used to ascertain the association (attachment and/or uptake) of PS particles in undifferentiated Caco-2 cells. In Caco-2 cells, the internalization of both 50 and 500 nm PS particles increased as particle concentrations increased, as seen in Figure 2A,B. However, 1 µm PS particles appeared to not be taken up by Caco-2 cells at any concentration (Figure A1). The percentage of Caco-2 cells that took up 50 nm particles were 0%, ~6%, and ~89% at 1, 10, and 100 µg/mL, respectively (Figure 2B). Meanwhile, 500 nm particles were taken up in ~2% of cells at 1 µg/mL, ~10% at 10 µg/mL, and ~51% at 100 µg/mL (Figure 2B). Cellular uptake was visualized using microscopy (Figure 2C).

#### 3.1.3. Clearance Assay

**Figure 3:**
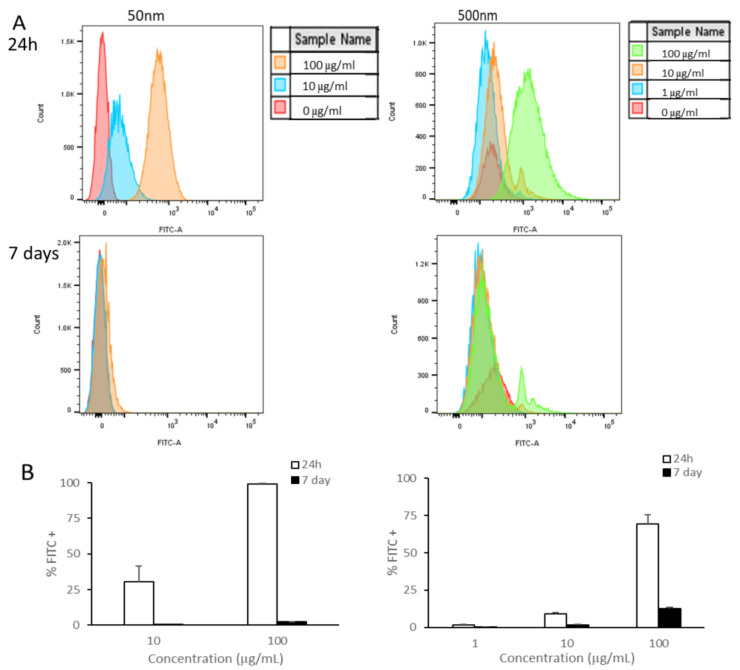
Caco-2 retention of PS-NPs and PS-MPs. Uptake and retention was analyzed by flow cytometry after undifferentiated Caco-2 cells were exposed to YG-PS-beads for 24 h and subsequently cultured for 6 days. (**A**) Representation of mean fluorescent intensity peaks of Caco-2 exposed to YG-PS-beads for 24 h and cultured for subsequent 6 days. (**B**) Quantification of percentage of Caco-2 that internalized YG-PS-NPs and -MPs after 24 h and retained PS-NPs and -MPs 6 days later (n = 3).

As shown, the smaller PS particles accumulated in cells after exposure for 24 h (Figure 2). Using flow cytometry, we examined if cells were able to remove these particles or if they were being retained by the cells. We observed a reduction in the amount of fluorescent signal in cells cultured for a subsequent 6 days in PS particle-free media after 24 h of exposure to PS particles (Figure 3). Remarkably, Caco-2 cells treated with 50 nm PS-NPs showed a clearance of >95% (Figure 3B). In contrast, Caco-2 cells retained <15% of the 500 nm beads at the highest concentration (Figure 3B).

### 3.2. Differentiated Transwell Mono-Culture

#### Barrier Integrity and Translocation

**Figure 4:**
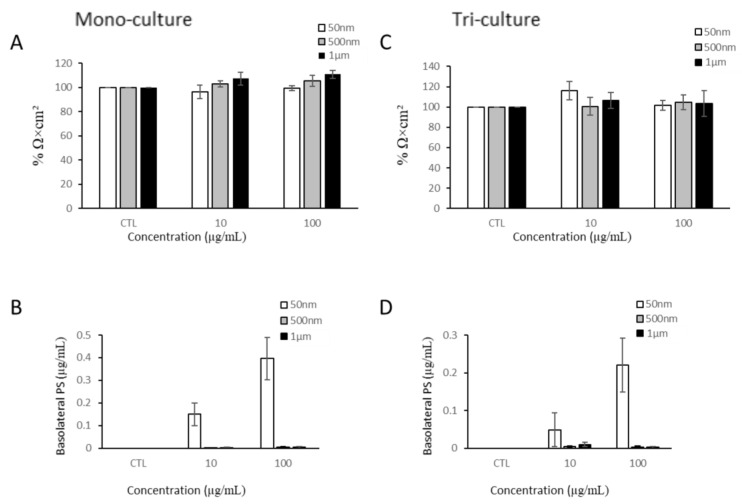
Barrier integrity of mono-culture and co-culture after PS-NP and PS-MP exposure. (**A**) Transepithelial electrical resistance of differentiated Caco-2 mono-culture after 24 h of NP/MP exposure normalized to control (CTL). (**B**) Quantification of translocation of YG-PS-beads through Caco-2 monolayer. (**C**) Transepithelial electrical resistance of Caco-2/HT29-MTX/THP-1 co-culture after 24 h of NP/MP exposure normalized to control. (**D**) Quantification of translocation of YG-PS-beads through Caco-2/HT29-MTX monolayer. Data are mean ± SEM from four independent experiments (n = 4). One-way ANOVA with Dunnet’s post hoc test was used with *p* > 0.05 indicating N.S.

The intestinal barrier is one of the primary locations for NP/MP accumulation and exposure. Thus, we investigated the impacts of NP/MP exposure on barrier integrity using TEER measurements and particle translocation. Since 1 µg/mL of PS-NPs and PS-MPs showed little to no uptake, we opted to test with the two highest doses, 10 and 100 µg/mL. After 24 h of exposure to all three PS particle sizes, no significant changes in the TEER values were detected (Figure 4A). Furthermore, the translocation of particles across the cellular monolayer was quantified by measuring the fluorescent signal in the basolateral compartment. Only 50 nm PS-NPs were able to cross both membranes into the basolateral compartment, reaching a maximum of 0.39 µg/mL (Figure 4B).

### 3.3. Transwell Tri-Culture

#### 3.3.1. Barrier Integrity and Translocation

**Figure A2:**
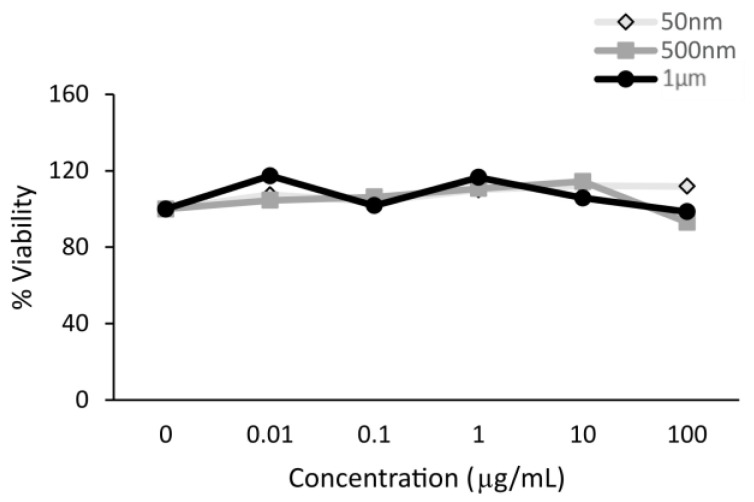
Polystyrene particles caused no cytotoxicity in HT29-MTX-E12 cells. Cell viability was assessed by an MTT assay in cells exposed to polystyrene particles (50 nm, 500 nm, and 1 µm) at concentrations of 0–100 µg/mL for 24 h (n = 1).

After evaluating the effects of the NP/MPs in the Caco-2 mono-culture, we applied NP/MPs to the apical side of a tri-culture model. In order to rule out any potential effects due to the addition of HT29-MTX-E12 cells to the apical side of the transwell, we assessed the viability of the HT29 cells after 24 h of MP exposure (Figure A2). The Caco-2 and HT29 cells were co-cultured on the apical side of the transwell membrane, while PMA-differentiated THP-1 cells were established at the bottom of the well of the basolateral compartment. Similarly to the mono-culture, exposure to PS particles for 24 h did not decrease the TEER values (Figure 4C). However, the addition of the mucus produced by the HT29-MTX-E12 cells attenuated the amount of 50 nm PS-NPs that were able to translocate across the cellular barrier by at least two folds (Figure 4D).

#### 3.3.2. Mucus Formation

**Figure 5:**
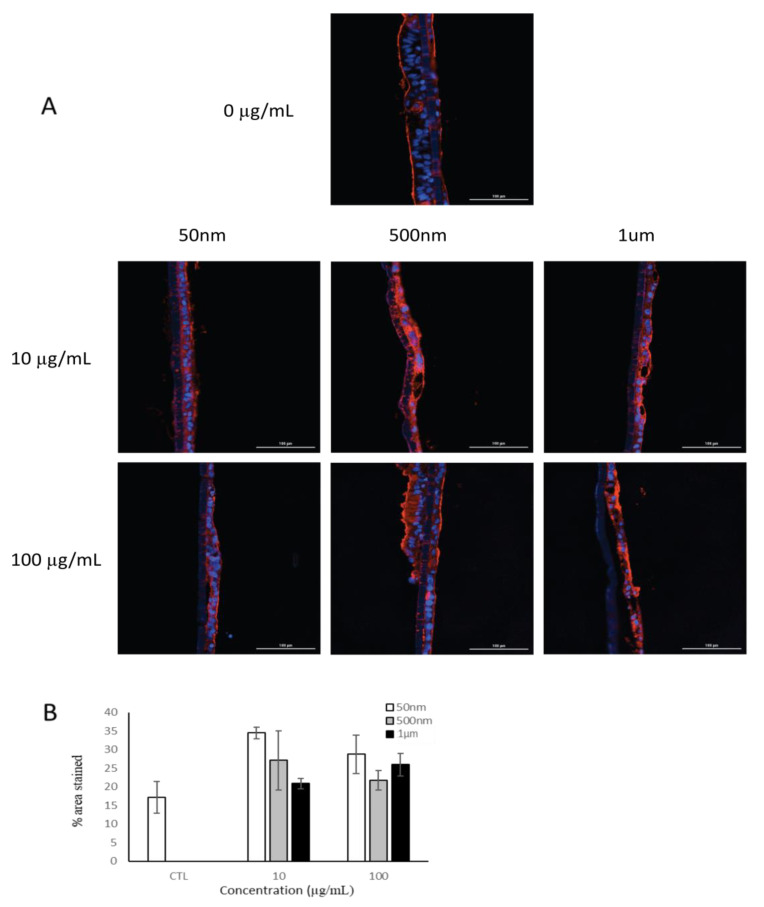
Fluorescent microscopy of mucus layer in tri-culture model after 24 h of exposure to PS particles (n = 1). (**A**) Cell nuclei were stained with DAPI (blue) and mucus (red) was stained with wheat-germ agglutinin. (**B**) Quantification of mucus staining as measure of mucus production. Data are mean ± SEM.

To investigate the possible effects of PS particle exposure on mucus production, mucus was stained with wheat-germ agglutinin (Figure 5). Our results showed that there was no reduction in the mucus layer due to PS particle exposure after 24 h (Figure 5B).

#### 3.3.3. THP-1 and Barrier Integrity

**Figure 6:**
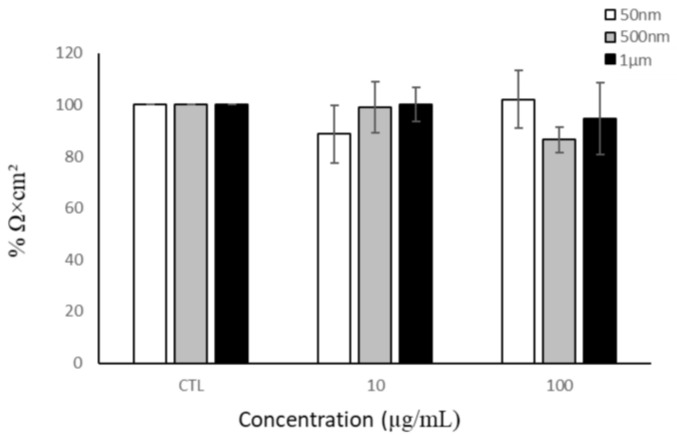
Barrier integrity of Caco-2/HT29-MTX monolayer after THP-1 basolateral exposure to NPs/MPs. Transepithelial electrical resistance after 24 h of NP/MP exposure normalized to control. Data are mean ± SEM from four independent experiments (n = 4). One-way ANOVA with Dunnet’s post hoc test was used with *p* > 0.05 indicating N.S.

As particles have been shown to translocate across the intestinal barrier, they could potentially activate intestinal macrophages, which may further produce factors that impact barrier integrity. Therefore, we examined the impact of THP-1 cells on barrier integrity. We added 50 nm, 500 nm, and 1 µm PS particles to the basolateral compartment of the tri-culture model. After 24 h of exposure, the TEER values were measured. THP-1 cells exposed to NP/MP particles for 24 h had no significant impact on the Caco-2/HT29-MTX monolayer integrity (Figure 6).

## 4. Discussion

As plastic production and, with it, plastic pollution increase, humans may be exposed to nano- and microplastic particles [2,67]. Consequently, more information is required to evaluate their potential effects on human health. The number of studies using human cells or mammalian models to evaluate the biological effects associated with MNPL exposure is quickly growing and disparities can arise from the various cell models used, diverse methodologies, and the different sizes, shapes, or compositions of plastic particles [68,69]. As they are of interest, cells representing potential exposure targets should be investigated first [52]. Hence, we investigated the effects of NP and MP exposure on intestinal cells.

By analyzing and comparing increasingly complex models of in vitro intestinal cell cultures, we are able to eliminate some of the variability in assessing toxicity across model type, while still maximizing the advantage of each model for particular endpoints and purposes. Herein, particle size, polymer type, and exposure dose were consistent across each model tested. In this study, we investigated the cellular uptake, toxicity, barrier integrity, and particle translocation of micro- and nano-PS particles (50 nm, 500 nm, and 1 µm) at various concentrations in three intestinal cell models. In the undifferentiated Caco-2 model, our results showed that exposure to PS-NP and PS-MP particles for 24 h did not elicit any apparent cytotoxicity. Our findings align with similar studies which have observed no or very mild cytotoxicity in Caco-2 cells exposed to PS-NP particles (50–100 nm, 200 nm) or (1, 2, 4, 5, and 10 µm) PS-MP particles at various concentrations for different exposure times, ranging from 1 to 72 h [14,52,58,69,70,71,72]. However, a few studies show contradictory results. Wu et al. (2020) detected no change in the cell viability of Caco-2 cells treated with 5 µm MPs up to 100 µg/mL after 24 h but saw a 10% decrease in viability after 48 h [73]. Xu et al. (2021) saw no changes in the number of Caco-2 cells treated with 100 nm NPs for 24 h yet observed toxicity at the highest dose after 48 h of exposure and at all doses after 96 h of exposure [74]. Interestingly, 100 nm PS particles caused damage to the plasma membrane of the Caco-2 monolayer after 96 h of exposure while 5 µm particles did not [75]. After 24 h, 1–1.9 µm PS-MPs decreased Caco-2 cell viability in a concentration-dependent manner and the photo-transformation of the PS-MPs exacerbated this effect [76]. With larger 10 and 100 µm PS-particles, Ding et al. (2024) noted increased LDH activity after 48 h of exposure in Caco-2 cells but observed no changes with smaller PS particle sizes of 0.1 and 1 µm [77].

In other gastrointestinal cell models, a diverse effect on toxicity has been reported. PS-NP particles differentially altered cell viability based on size in human gastric adenocarcinoma (AGS) cells; 44 nm particles decreased cell viability at 10 µg/mL, while 100 nm particles increased viability [78]. Ding et al. (2021) observed that 60 nm NPs at 50 µg/mL decreased cell proliferation and increased apoptosis in human gastric epithelial (GES-1) cells after 24 h [79]. In a human colon cancer cell line (HT-29), 3 and 10 µm PS-MPs had a moderate cytotoxic effect in a dose-dependent manner at 100–1600 particles/mL [80]. In human colonic epithelial cells (CCD841CoN) and small intestinal epithelial cells (HIEC-6), no effect on cell viability was noted after 24 h of treatment with PS-MPs (0.1, 0.5, 1, and 5 µm) at concentrations ranging from 12.5 to 100 µg/mL [81]. CCD-18Co human intestinal cells showed no cytotoxicity 48 h after treatment with 0.5 µm and 2 µm carboxylated PS particles up to 20 µg/mL [82], but smaller, neutral 50 nm and 100 nm PS particles decreased cell viability > 10% after 48 h of treatment with 10 µg/mL [83]. Moreover, a comprehensive study using SNU-1 human gastric epithelial cells treated with a wide range of NP/MPs (50, 100, 200, 500, 1, and 5 µm) for 1–24 h at concentrations of 0.1–100 µg/mL was performed by Banerjee et al. (2021). Markedly, PS particles were reported to be toxic at both the smaller and larger end of the size spectrum tested [84].

Interestingly, in various co-culture models that included intestinal enterocytes, mucus-secreting goblet cells, and lymphoblast-like cells, Domenech et al. (2020) showed that no cytotoxicity was observed after 24 h of treatment with 0.05–0.1 µm nanoparticles and DeLoid et al. (2021) saw no changes in cytotoxicity after exposure to 25 nm and 1 µm PS particles at 400 and 1000 µg/mL for 24 h [64,85]. Lehner et al. (2020) observed no cytotoxicity in a co-culture model exposed to MPs > 70 µm but less than 300 µm for 48 h [86]. Furthermore, Busch et al. (2021), using a tri-culture model (Caco-2/HT29-MTX-E12/THP-1), did not observe any effects regarding cytotoxicity, DNA damage, barrier integrity, or cytokine release after 24 h of treatment with PS particles [70].

Micro- and nanoplastic exposure is likely to be continuous [69]; therefore, we examined the effects of nano- and microplastics with a prolonged exposure of 7 days. Undifferentiated Caco-2 cells showed no changes in cell viability after 7 days of exposure to MNPLs. Similarly, in other long-term experiments on human intestinal cells (Caco-2 and HT-29) using 50 nm, 3 µm, and 10 µm PS particles, no detrimental effects of microplastic exposure over 48 days of exposure were observed [69,80].

Despite the lack of cytotoxicity, we confirmed that Caco-2 cells were able to internalize both 50 nm and 500 nm PS particles but not 1 µm PS particles. Using fluorescent YG-PS particles allowed for the visualization of particle uptake in individual cells. PS-NPs (50 nm) were not detected until 10 µg/mL, while PS-MPs (500 nm) were detected starting at 1 µg/mL. However, at the highest concentration (100 µg/mL), 50 nm PS-NPs were more readily taken up than 500 nm particles. Multiple studies have confirmed the uptake of numerous different particles sizes in a plethora of cell lines including intestinal epithelial, gastric epithelial, hepatocyte, and placental endothelial cells [2,4,50,52,58,64,69,74,78,79,82,84,87,88,89,90].

MP and NP uptake is well documented, but the fate of these particles is not well understood. Internalization is dependent on particle size, cell type, and the cellular transport mechanism [91]. MNPLs can be internalized through active endocytosis and passive penetration and can be excreted via energy-dependent and -independent pathways [79,91]. Caco-2 cells showed a reduction in the amount of fluorescent signal in cells cultured for 6 days after 24 h of exposure. It is of interest that cells treated with 50 nm PS-NPs showed a clearance of >90%, while cells treated with 500 nm PS-MPs retained some of the particles at all concentrations. The reduction in the fluorescent signal, indicating a decrease in intracellular particles, was in part due to cell division and exocytosis [91]. The retention of the larger particles (500 nm) was indicative that the release of MNPLs was size-dependent and perhaps that the larger particles required more energy-dependent release. These results have been described before by Liu et al. (2021) in rat basophilic leukemia cells (RBL-2H3), where 50 nm PS particles were shown to be cleared from the cells more readily than 500 nm PS particles [91].

Since ingestion is one route of MNPL exposure and smaller particles may pass through the gut barrier [5,33,35,40,47,48,49,67,69], we further examined a more complex cell model using a mono-culture and tri-culture transwell system to investigate impacts on barrier integrity. The mono-culture model, using differentiated Caco-2 cells, showed no changes in barrier integrity after 24 h of exposure to MNPLs of any size at all concentrations tested. Additionally, the tri-culture model also exhibited no changes in the TEER values after 24 h of exposure to 50 nm, 500 nm, and 1 µm PS particles. Comparably, Busch et al. (2021) observed no changes in TEER values in a tri-culture model 24 and 48 h post-exposure to 50 nm PS particles [70]. Also, no changes in barrier integrity were noted in Caco-2/HT29 co-cultures exposed to either 0.04–0.09 µm PS particles or 50 nm and 500 nm COOH-modified PS particles for 24 h [50,64]. In addition, Walczak et al. (2015) noted no changes in TEER values for either mono-cultures or co-cultures of Caco-2 cells exposed to 50 nm and 100 nm PS particles for 24 h at 250 µg/mL [40]. In contrast, Ding et al. (2024) observed a reduction in TEER values, indicating the degradation of the monolayer, in Caco-2 cells exposed to 0.1, 1, 10, and 100 µm PS particles for 6 h at 500 µg/mL [77].

Translocation across the gut barrier is of great interest, as it may allow for the systemic distribution of MNPLs [5,33,35,40,47,48,49]. Our findings show that 50 nm PS-NPs were the only size able to translocate across the Caco-2 monolayer, but the presence of mucus in the tri-culture model attenuated this. The in vivo mucus layer is substantially thicker and would further attenuate particle translocation [92]. A study by Walczak et al. (2015) demonstrated that 50 nm and 100 nm PS-NPs were able to cross the monolayer of a Caco-2 mono-culture and Caco-2/HT29 co-culture after 24 h [40]. Additionally, 50 nm and 200 nm carboxylated PS particles were found to cross the monolayer of a Caco-2/HT29 co-culture after 45 min of exposure [93]. However, Hesler et al. (2019) detected no translocation of 50 nm or 500 nm COOH-modified PS particles across a Caco-2/HT29 co-culture after 24 h [50]. The layer of mucus is the first level of defense in the gut and its absence can be detrimental [94]. In the tri-culture model, we observed that production of mucus was not reduced after 24 h of PS particle exposure. These results are in line with other findings that did not see a change in mucus staining patterns after exposure to 50 nm PS particles [70]. Additionally, macrophages are immune defense cells that respond to signals in the microenvironment, regulating an inflammatory response [95]. We investigated whether THP-1 cells exposed to MNPLs created an inflammatory response that would impact intestinal barrier integrity. No change in barrier integrity was detected after 24 h of NP/MP exposure to THP-1 cells.

Limited data availability on the presence of micro- and nanoplastics in the human diet means that uncertainties remain in regard to their potential exposure levels, absorption, and distribution to various tissues [3]. A caveat of current research is the absence of standardized methods and analytical techniques for measuring nano- and microplastics in environmental matrices, including foods. Therefore, additional research is needed to determine environmentally relevant concentrations of microplastics, thus allowing for future toxicity studies with relevant test materials and exposure levels to characterize potential risk to human health and conduct proper risk assessments. Moreover, human exposure may be dependent on a variety of external factors including exposure routes, local geography, local weather patterns, predominant particle size, surface chemistry, and local microplastic milieu composition.

## 5. Conclusions

Overall, our study shows that NPs and MPs had no detrimental effect on intestinal cells at concentrations up to 100 µg/mL, despite particle uptake. Both NP (50 nm) and MP (500 nm) particles were readily internalized by Caco-2 cells following exposure for 24 h, but MPs were more readily retained inside the cells compared to NP particles. Additionally, MP/NP exposure did not impact barrier integrity. This study reduced some of the variability common to MNPL testing by exposing multiple cell culture systems to MNPLs of the same particle size, type, and concentration. By exploiting the benefits of each culture system, we were able to perform a comprehensive analysis of MP/NP exposure on in vitro intestinal models.

Furthermore, exposure to MNPLs is likely to be continuous and varied; hence, further studies using a variety of plastic particles with different sizes, shapes, and polymer types are needed to elucidate the potential effects of human MNPL exposure.

## Data Availability

Data will be made available upon request.

## Abbreviations

The following abbreviations are used in this manuscript:

MPs	Microplastics
NPs	Nanoplastics
MNPL	Micro- and nanoplastic
GIT	Gastrointestinal tract
TEER	Transepithelial electrical resistance

## Appendix A

**Table A1:** PS-NP and -MP characterization.

	50 nm		500 nm		1 µm	
	Distilled H_2_O	Media	Distilled H_2_O	Media	Distilled H_2_O	Media
Size (nm)	67.2 ± 0.22	98 ± 0.48	533 ± 11.24	700 ± 7.25	800 ± 20.74	900 ± 20.62
PdI	0.02 ± 0.01	0.1 ± 0.01	0.03 ± 0.02	0.11 ± 0.04	0.2 ± 0.09	0.6 ± 0.10
